# Do Implicit Theories About Ability Predict Self-Reports and Behavior-Proximal Measures of Primary School Students’ In-Class Cognitive and Metacognitive Learning Strategy Use?

**DOI:** 10.3389/fpsyg.2021.690271

**Published:** 2021-06-22

**Authors:** Benjamin Matthes, Heidrun Stoeger

**Affiliations:** Chair for School Research, School Development, and Evaluation, Institute for Educational Science, Faculty of Human Sciences, Universität Regensburg, Regensburg, Germany

**Keywords:** implicit theories about ability, mindsets, incremental theory, entity theory, growth mindset, fixed mindset, cognitive learning strategies, metacognitive learning strategies

## Abstract

Although studies show relations between implicit theories about ability (ITs) and cognitive as well as metacognitive learning strategy use, existing studies suffer from an overreliance on broad-brush self-report measures of strategy use and limited ecological validity. Moreover, studies rarely examine younger students, and research on ITs and how much students benefit from interventions on learning strategies is lacking. Therefore, we investigated in ecologically valid settings (regular classroom instruction) whether primary school students’ ITs are related to their use of cognitive strategies (text reduction strategies based on identifying a text’s main ideas) and metacognitive strategies, assessed with (a) typical self-report scales and (b) more behavior-proximal measures. We also investigated whether students’ ITs predict how much they benefit from a previously evaluated 4-week intervention on cognitive and metacognitive strategies during regular classroom instruction (i.e., how much self-report scales and behavior-proximal measures for strategy use increase over the course of the intervention). Participants were 436 German primary school students (third and fourth graders). The data were analyzed using mixed linear regression analyses. Strength of students’ incremental theory was positively related to metacognitive strategy use, but not cognitive strategy use, when measured with self-report scales. For behavior-proximal measures, strength of incremental theory was positively related to the effectiveness of students’ cognitive strategy use and their extent of strategy monitoring (one of the two metacognitive strategies examined), but not to the quality of their goal setting (the second metacognitive strategy). Unexpectedly, students with a stronger incremental theory did not benefit more from the intervention.

## Introduction

While some learners believe that their abilities can be greatly increased through practice, others believe that their abilities have a large static part that cannot be changed. Such beliefs, known as implicit theories about ability (ITs; see Dweck, 2000), have well-documented influences on learning and achievement behavior (see Burnette et al., 2013). For example, learners who believe that their abilities can be greatly increased tend to appreciate learning (Dickhäuser et al., 2016) as well as challenges and effort (Lin-Siegler et al., 2016), and to see setbacks as learning opportunities (e.g., Smiley et al., 2016).

An important aspect of learning that seems to be related to ITs but that has not been comprehensively investigated is learning strategy use. Of interest here are both cognitive learning strategies (techniques that enhance information processing; Zeidner and Stoeger, 2019) and metacognitive learning strategies (techniques related to the metacognitive processes of goal setting, planning, self-monitoring, self-control, and self-evaluation; Dent and Koenka, 2016).

Existing studies on ITs and the use of cognitive and metacognitive learning strategies exhibit several weaknesses. First, they typically rely on broad-brush self-report measures. That is, these studies use items that ask learners about the extent to which they use particular strategies for learning in general or for a given subject or class, thereby requiring learners to generalize over a variety of learning episodes and contexts (e.g., Martin et al., 2013; Mega et al., 2014). This can be problematic due to the validity issues of such measures (see Schellings and van Hout-Wolters, 2011; Veenman, 2011a,b).

Second, even among the studies that used more behavior-proximal measures than broad-brush self-report items asking about the extent of learners’ strategy use (Wood and Bandura, 1989; Tabernero and Wood, 1999; Greene et al., 2010; Beckmann et al., 2012; Karlen and Compagnoni, 2017), the majority has limited ecological validity. Most of these studies were implemented in somewhat artificial contexts, with many employing laboratory tasks far removed from academic learning (Wood and Bandura, 1989; Tabernero and Wood, 1999; Beckmann et al., 2012).

Third, only few studies have examined younger students—and among these, all we are aware of have used broad-brush self-report scales to assess strategy use (Stipek and Gralinski, 1996; Law, 2009). This can be considered a research gap because investigating predictors of learning strategy use seems particularly interesting among younger students—who are an important target group for learning strategy instruction (Dignath et al., 2008).

These weaknesses of existing research call for further studies on this topic. But not only the relationships between ITs and cognitive and metacognitive strategy use are educationally relevant: It is equally important to investigate whether students’ ITs influence how much they benefit from learning strategy interventions. However, to the best of our knowledge, this has not been investigated yet. Based on related research, we assume that students with more incremental beliefs should be more open to instruction in the use of learning strategies and benefit more from it. For example, incremental theorists tend to be more focused on increasing their competencies than entity theorists (e.g., Robins and Pals, 2002; Dupeyrat and Mariné, 2005; Lin-Siegler et al., 2016; see also Burnette et al., 2013), as well as more likely to avail themselves of learning opportunities (Hong et al., 1999; Nussbaum and Dweck, 2008).

Based on the abovementioned weaknesses and research gaps, our first aim was to investigate whether ITs predict the use of cognitive and metacognitive learning strategies of primary school students in an ecologically valid setting. In addition to typical broad-brush self-report scales, we included behavior-proximal measures of strategy use that were collected while students worked with authentic learning materials (expository texts) on a daily basis over the course of 1 school week.

Our second aim was to investigate whether and to what extent students’ ITs predict how much they benefit from a 4-week intervention on cognitive and metacognitive strategies in their regular classroom context. We employed an intervention whose effectiveness had been demonstrated in an evaluation with a pre–post–follow-up control-group design (Stoeger et al., 2014) and analyzed whether students’ ITs predict increases in strategy use and its effectiveness (self-reported and measured in a more behavior-proximal manner) when students continue to work on daily expository texts and receive feedback.

## Theoretical Background

### Cognitive and Metacognitive Learning Strategies

Cognitive learning strategies are techniques directly related to the accomplishment of a cognitive task (Alexander et al., 1998)—for example, the task of understanding the main ideas of an expository text about a scientific topic—and that enhance information processing (Zeidner and Stoeger, 2019). Because we investigated primary school students, we focused on cognitive strategies in the service of text reduction and the identification of a text’s main ideas, which are essential for reading comprehension (Gajria and Jitendra, 2016) and therefore important from primary school on (Williams, 1988). Two effective cognitive strategies based on main idea identification are summarizing and mapping (see Fiorella and Mayer, 2016). Summarizing requires learners to locate a text’s most essential pieces of information, to compress them into a short form, and to reformulate them in their own words (Westby et al., 2010). Mapping requires learners to spatially arrange a text’s most essential pieces of information and to establish connections between them (Fiorella and Mayer, 2016), resulting in a graphical representation such as a concept map or mind map. Both summarizing and mapping depend on learners’ ability to identify main ideas (Westby et al., 2010; Leopold and Leutner, 2015), making correct main idea identification a prerequisite for these strategies’ effective application.

Metacognitive learning strategies refer, in the broadest sense, to the use of skills to control one’s cognitive processes (Efklides, 2008) in service of regulating one’s learning. Although several theoretical approaches exist that focus on different metacognitive processes (see Panadero, 2017, for an overview), there is some agreement on the key processes of goal setting, planning, self-monitoring, self-control, and self-evaluation (Dent and Koenka, 2016). Among these, goal setting is especially important due to its role in guiding subsequent metacognitive processes (Dent and Koenka, 2016). Specific and challenging goals serve as a standard for self-evaluation and provide feedback regarding the effectiveness of one’s learning when combined with systematic self-monitoring (Zimmerman, 2008), which is another key metacognitive strategy (Zimmerman and Paulsen, 1995). Such metacognitive monitoring involves learners tracking their learning, its results, and its effectiveness (Zimmerman and Moylan, 2009), thereby enabling them to make the necessary changes to achieve their goals (Zimmerman and Paulsen, 1995).

### Implicit Theories About Ability and General Approaches to Learning

Implicit theories (also called mindsets; Lüftenegger and Chen, 2017) are lay theories about the nature of traits and abilities (Molden and Dweck, 2006) that affect how learners approach potential learning situations (see Dweck and Master, 2008). Two theories can be distinguished (Dweck and Leggett, 1988; Dweck, 2000): an incremental theory (or growth mindset)—the belief that traits and abilities can be fundamentally changed, and an entity theory (or fixed mindset)—the belief that traits and abilities contain a large unchangeable part. These two theories are often understood as opposite ends of a bipolar continuum (e.g., Ehrlinger et al., 2015; Haimovitz and Dweck, 2016).

Whereas those who hold an incremental theory tend to believe that abilities can be greatly improved, those who hold an entity theory tend to believe that abilities have a large static component that cannot be improved (see Dweck and Master, 2008). Therefore, for incremental theorists, performance outcomes provide information about how to improve one’s abilities (Mangels et al., 2006), whereas for entity theorists, performance outcomes provide information about the extent of one’s fixed abilities (Rattan et al., 2012). Consequentially, incremental theorists tend to focus on learning and often want to improve their abilities by overcoming challenges (Chen, 2012; Dickhäuser et al., 2016), while entity theorists tend to focus on appearing competent, even at the expense of learning (Martin et al., 2013). Whereas incremental theorists tend to believe that setbacks indicate insufficient effort (Hong et al., 1999; Smiley et al., 2016) and that the need to invest effort signifies an optimally challenging learning situation (Miele et al., 2013; Lin-Siegler et al., 2016), entity theorists tend to believe that setbacks indicate stable deficits (Martin et al., 2001) and that the need to invest effort implies low ability (Baird et al., 2009; Tempelaar et al., 2015). Therefore, the more learners hold an incremental theory, the more effort they tend to invest (Cury et al., 2008; Ziegler and Stoeger, 2010; Mouratidis et al., 2017), and the more adaptively they react to setbacks. Whereas incremental theorists tend to look for ways to remedy their deficits (Hong et al., 1999; Ziegler et al., 2010a; Dresel et al., 2013) and to increase their effort (Jones et al., 2012; Rickert et al., 2014), entity theorists tend to experience negative affect (Shih, 2011), to reduce their effort (Smiley et al., 2016), and to consider giving up (Robins and Pals, 2002).

### Implicit Theories and Learning Strategies

Based on this, it can be hypothesized that individuals with a more incremental theory are more likely to use (cognitive and metacognitive) learning strategies due to their more learning-oriented outlook (see Dweck and Master, 2008). There are also several studies that confirm these relations.

#### Implicit Theories and Learning Strategy Use

##### Studies Employing Broad-Brush Self-Report Measures

Several investigations with adults show that learners with more of an incremental theory tend to report using both more cognitive and metacognitive strategies. Bråten and Olaussen (1998) found that endorsing more of an incremental theory about intellectual qualities predicted greater scores on a self-report scale for both cognitive and metacognitive strategy use. In a study by Martin et al. (2001), learners with stronger incremental theories about scholastic abilities reported more metacognitive strategy use. Vermetten et al. (2001) found learners with greater incremental beliefs about intelligence to score higher on most of their self-report scales for cognitive strategy use, but not on the one for metacognitive strategy use. In a study by Dupeyrat and Mariné (2005), agreement with incremental theory items about intelligence was related to reporting more cognitive strategy use, while agreement with entity theory items was unrelated to strategy use. Mega et al. (2014) found that incremental beliefs about intelligence predicted higher scores on several self-report scales measuring the use of different cognitive and metacognitive strategies. In a study by Stump et al. (2014), endorsing an incremental theory about intelligence was related to greater self-reported cognitive strategy use. Yan et al. (2014) found that learners with an incremental theory about intelligence reported more metacognitively sophisticated studying habits than learners with an entity theory. Finally, a study by Karlen and Compagnoni (2017) found that an incremental theory about writing ability predicted higher scores on most of their scales assessing metacognitive writing strategies.

Similar results were obtained in studies with high school students. Ommundsen (2003) found students with more incremental beliefs about athletic abilities to report using more cognitive and metacognitive strategies in physical education. In a study by Ommundsen et al. (2005), agreement with incremental theory items about academic ability predicted higher scores on a self-report scale about cognitive and metacognitive strategy use; agreement with entity theory items, however, was unrelated to strategy use. Martin et al. (2013) found that more incremental beliefs about intelligence were related to higher scores on a self-report scale for learning strategy use that focused on metacognitive strategies.

The two investigations with younger students we are aware of have also obtained comparable results. In a study by Stipek and Gralinski (1996), students from grades 3 to 6 scored higher on a self-report scale for cognitive and metacognitive strategy use the more they agreed to incremental theory items about ability; agreement to entity theory items, however, was unrelated to the strategy scale. Law (2009) found that the more primary school students held an incremental theory about reading ability, the higher they scored on a self-report scale for cognitive and metacognitive reading strategy use.

##### Studies Employing Behavior-Proximal Measures

However, few of the investigations about how ITs relate to cognitive and metacognitive strategy use have used behavior-proximal measures of strategy use. Even the few studies that did so employed learning situations that were laboratory tasks with little resemblance to academic learning—and all of them were conducted with adult learners. The study by Wood and Bandura (1989) featured a management simulation consisting of three trials. Compared to participants who had received an entity theory manipulation (about decision-making ability), those who had received an incremental theory manipulation set more challenging performance goals throughout the trials. Tabernero and Wood (1999) had their participants work on a 90-min computer-based management simulation. Compared to participants who held an entity theory about the ability to manage work groups, participants who held an incremental theory set more challenging goals for their performance from the beginning. In a similar study by Beckmann et al. (2012), participants worked in groups of three on a computer-based management simulation with two blocks of trials. Each group consisted of either three persons with an incremental theory about managerial ability or of three persons with an entity theory. The groups set goals for their performance before the first block of trials, before the second block, and after the second block. Compared to entity theorist groups, the incremental theorist groups set more challenging goals both before and after the second block.

We are aware of only two studies that have investigated how ITs relate to behavior-proximal measures of strategy use in situations that one might encounter in the context of academic learning. In both studies, the participants were adults, and both studies reported null results. Greene et al. (2010) gave their participants 30 min to complete a learning task in a hypermedia environment. While working, participants verbalized their thoughts. Afterwards, the self-regulated learning activities mentioned by the participants were counted. The learning activities under investigation mainly represented either cognitive or metacognitive strategies. Surprisingly, values for self-regulated learning activities were unrelated to how strongly participants held an incremental theory about intelligence. In a study by Karlen and Compagnoni (2017), participants had to answer open-ended questions about what they did before, during, and after writing an academic essay. The quality of participants’ metacognitive strategy use was rated based on these responses. Unexpectedly, there was no relationship between participants’ incremental beliefs about writing ability and the quality of their metacognitive strategies.

The studies on ITs and cognitive as well as metacognitive strategy use show that this research area suffers from an overreliance on broad-brush self-report scales. Despite the criticism that such measures have received (see Schellings and van Hout-Wolters, 2011; Veenman, 2011a,b), only few of the aforementioned studies employed behavior-proximal measures of strategy use—and almost all of the studies that did were conducted in somewhat artificial situations far removed from academic learning, which limits their ecological validity. Another limitation is that most studies were conducted with adult learners. This tendency is particularly pronounced among those studies that employed behavior-proximal measures of strategy use.

#### ITs and the Use of Learning Strategy Interventions

Although it seems plausible that ITs might predict how much learners make use of interventions on learning strategies—that is, benefit from them—to the best of our knowledge, no studies exist on this topic. However, there is some indirect evidence. First, incremental theorists tend to be more strategic about their learning than entity theorists (see Dweck and Master, 2008), which is also reflected in their aforementioned tendency to report using more cognitive and metacognitive strategies. Thus, incremental theorists should also be more open to instruction in the use of such strategies. Second, with respect to learning situations in general, incremental theorists tend to be more focused on increasing their competencies than entity theorists (e.g., Dupeyrat and Mariné, 2005; Lin-Siegler et al., 2016; see Burnette et al., 2013) and more likely to avail themselves of learning opportunities (Hong et al., 1999; Nussbaum and Dweck, 2008). Thus, incremental theorists should also make more use of interventions that give them the opportunity to practice cognitive and metacognitive strategies. In other words, they should show greater increases in their amount of learning strategy use and its effectiveness over the course of such an intervention.

### The Present Study

The first aim of this study was to investigate how primary school students’ ITs relate to their use of cognitive and metacognitive learning strategies. To replicate previous findings obtained with older learners, we employed (a) typical self-report scales on cognitive and metacognitive learning strategy use. To broaden existing research, we employed (b) behavior-proximal measures of these strategies. In contrast to most existing studies, we analyzed these relations in authentic academic learning situations (regular classroom instruction) among primary school students. In addition to employing typical self-report scales, we investigated the baseline levels for the usage of cognitive and metacognitive strategies *via* behavior-proximal measures after the strategies had been introduced by the students’ teachers. For 1 week (the baseline week), students tried to extract the 10 main ideas from one expository text per school day by using the text reduction strategies that had been introduced to them. In addition, they set goals and monitored their learning with the help of their learning diaries.

The second aim was to investigate how ITs relate to the extent to which primary school students make use of a previously evaluated 4-week intervention in which the strategies introduced before the baseline week were proceduralized. In particular, we investigated how the measures of cognitive and metacognitive strategy use (self-report measures and behavior-proximal measures) changed over the course of the 4-week intervention (the proceduralization weeks) that followed after the baseline week.

We examined students at the end of primary school (grades 3 and 4 in Germany) because learning strategies can and should be taught as early as primary school (Dignath et al., 2008), in part because of their increasing importance in secondary school (see Dent and Koenka, 2016). Therefore, it seems worthwhile to examine possible predictors for the use of learning strategies and learning strategy interventions in this age group. However, because ITs as well as related beliefs and behaviors are still taking shape during this developmental stage (see Dweck, 2002; Barger and Linnenbrink-Garcia, 2016), we were not sure whether a positive relationship already exists between holding an incremental theory and learning strategy use. Although it appears that some of the negative effects of holding an entity theory are already evident in this age group (Kinlaw and Kurtz-Costes, 2007; Haimovitz et al., 2011), the consolidation of the related network of beliefs and behaviors is thought to continue into early adolescence (Dweck, 2002; Haimovitz et al., 2011). Also, students’ metacognitive skills are still in the process of developing at the end of primary school (see Veenman et al., 2006).

For the analyses related to both aims, we controlled for students’ reading comprehension. This was done because of the great role that reading plays in the task of identifying a text’s main ideas. Especially in this young age group, levels of reading comprehension might influence students’ performance on the task, their use of cognitive and metacognitive strategies, and the extent to which they make use of the intervention.

Our first prediction is that the more children hold an incremental theory, the greater their values will be on both self-report scales and behavior-proximal measures for both cognitive and metacognitive learning strategy use. Our second prediction is that the more children hold an incremental theory, the better use they will make of the 4-week intervention on learning strategies, that is, the more both the self-report scales for strategy use and the behavior-proximal measures for strategy use and effectiveness will increase over the intervention. Thus, in addition to expecting increases on all these measures, we expect that strength of incremental theory will be positively related to each measure’s growth rate.

## Materials and Methods

### Participants

The participants were 436 students (369 fourth graders and 67 third graders) from 20 classrooms of 19 primary schools in Bavaria, Germany. Data collection was part of a larger investigation involving teachers, parents, and students (see Matthes and Stoeger, 2018). We limited our analyses to those 20 classrooms (out of 24) in which the intervention had been implemented as intended.^1^ Our analyses included all students who had completed the implicit theory scale in the pre-intervention questionnaire, the reading comprehension test, and returned the learning diary introduced during the intervention program. Students’ average age was 9.7 years (ranging from 8 to 11 years, *SD* = 0.60) and 54% were girls. In 15% of the cases, either the student or one of their parents was born outside of Germany. Of the 372 students for whom information on parents’ educational level was available, 22% had at least one parent with a university degree.

### Measures

#### Predictors

##### Incremental Theory

Students’ ITs were assessed before the intervention with a modified version of the six-item scale from Ziegler and Stoeger (2010). The original items (that queried strength of incremental theory for the domain of mathematics) were modified to assess school-related domain-general ITs. The items were answered on a six-point Likert scale with response options from 1 (*completely disagree*) to 6 (*completely agree*). A sample item reads: “What I am capable of in school is not fixed. I can learn new things and expand my abilities.” The scale’s Cronbach’s alpha was 0.68.

##### Reading Comprehension

This covariate was assessed before the intervention with a shortened version of the first half of the two-part reading comprehension section of the Hamburg Reading Test for Grades 3 and 4 (HAMLET 3–4; Lehmann et al., 2006). This part originally contained 10 short texts, each followed by four multiple-choice questions where participants had to choose the correct answer from four alternatives. Our version included six of the 10 texts (texts 1, 3, 4, 5, 6, and 7) and the corresponding questions, that is, 24 of the 40 original questions. For each of these, participants received one point for selecting the correct answer, resulting in a total score of up to 24 points. Cronbach’s alpha for this total score was 0.77.

#### Self-Report Scales for Strategy Use

Self-report scales for cognitive and metacognitive strategies were completed before the baseline week and after the intervention.

##### Cognitive Strategies Scale

Cognitive strategies were assessed using a four-item scale that asked to what extent participants used text reduction strategies that are based on main idea identification (underlining and excerpting main ideas, drawing mind maps containing them, and writing summaries based on them). A sample item reads: “When I read a text, I underline the most important aspects.” Each item was answered on a six-point Likert scale ranging from 1 (*completely disagree*) to 6 (*completely agree*). Cronbach’s alpha was 0.70 for the first measurement point and 0.50 for the second measurement point. The low alpha value for the second measurement point was not unexpected, as students might have found out during the intervention which of the three strategies they had been introduced to was most helpful to them, and thus might have reported mainly using this strategy (and not the other two) at the second measurement point.

##### Metacognitive Strategies Scale

Metacognitive strategies were measured with a shortened, slightly adapted version of the Questionnaire for Self-Regulated Learning (FSL-7; Ziegler et al., 2010b). The original questionnaire measured six metacognitive strategies (self-assessment, goal setting, strategic planning, strategy monitoring, strategy adjustment, and outcome evaluation) for four school-related situations (studying for school, preparing for the upcoming school year during the summer holidays, preparing for an in-class test, and catching up on schoolwork after an illness). Each of these four situations came with one item for each of the six metacognitive strategies (i.e., 24 items altogether). Our scale contained three of these situations (preparing for the upcoming school year was not included) and thus consisted of 18 items. For each, a forced-choice format required participants to choose one of three responses. The first response indicated use of a metacognitive strategy to regulate the respective aspect of one’s learning (e.g., “When preparing for a test, I always set a specific goal as to what and how much I want to learn.”). The second response indicated reliance on teachers or parents for regulation (e.g., “The teacher or my parents tell me what goals I should set for myself when preparing for a test.”). The third response indicated disavowal of regulating the respective aspect (e.g., “When preparing for a test, I do not set any goals. I can fully rely on my intuition for this.”). Students’ values for this variable were determined by calculating the proportion of items for which they chose the response that represented metacognitive strategy use (relative to the number of items they had answered). The scale’s Cronbach’s alpha was 0.84 for the first measurement point and 0.93 for the second one.

#### Behavior-Proximal Measures of Strategy Use

Behavior-proximal measures for cognitive and metacognitive strategies were collected during the baseline week (to investigate the first aim) and during the four proceduralization weeks (to investigate the second aim).

##### Number of Correctly Identified Main Ideas

As an indicator for the effectiveness of cognitive strategy use, we used students’ reports on how many of the 10 main ideas from each day’s text they had correctly identified using the cognitive strategies (information which students recorded after they had corrected the respective text with their teacher). All texts were comparable regarding length and difficulty. During data entry, to ensure the validity of the students’ records regarding the number of correctly identified main ideas, a random sample of them was compared with the corresponding materials containing the main ideas that the students had identified. For our analyses, we averaged numbers of correct main ideas for the five texts of each week to reduce unsystematic variance. Cronbach’s alpha values for these five weekly averages ranged from 0.78 to 0.85.

##### Strategy Monitoring

The extent to which students used the metacognitive strategy of monitoring while working on the texts was measured with an item in their learning diary that read “I have monitored myself while using my strategy.” Students responded to this item each school day directly after having worked on the text—an approach that mitigates some of the problems that decontextualized, broad-brush self-report measures are prone to (see Veenman, 2011a). The item was answered on a six-point Likert scale from 1 (*completely disagree*) to 6 (*completely agree*). Again, the five values for each week were averaged. Cronbach’s alpha for these five averages ranged from 0.91 to 0.96.

##### Goal Setting, Operationalized as Deviation From Week’s Goal

To measure the effectiveness of students’ goal setting, we examined the deviation of their weekly goal (how many main ideas they aimed to identify in the week’s five texts) from the weekly average of their number of correctly identified main ideas. For this, we subtracted the week’s goal from the week’s average and calculated the result’s absolute value. Consequently, lower values represent more realistic goals. Students set their weekly goal by completing the sentence “My goal for this week is to find—out of 10 main ideas in the text” with a number from 1 to 10 at the beginning of the respective week.

### Procedure

First, students filled out the incremental theory scale and the self-report scales for cognitive and metacognitive strategy use. To facilitate understanding of the items, they were read aloud to the students by the respective teacher before the students answered them. Next, the students completed the reading comprehension test. Second, their teachers introduced cognitive and metacognitive learning strategies to the students. Next, teachers let the students try out these strategies during the baseline week. After that, the students took part in a 4-week learning strategy intervention, during which the strategies were proceduralized with the help of extensive feedback from their teachers (Stoeger and Ziegler, 2008). Finally, the students once again completed the self-report scales on their use of cognitive and metacognitive strategies.

During the introduction of the cognitive and metacognitive strategies, students were provided with declarative knowledge about these strategies during regular classroom instruction (for details, see Stoeger et al., 2014). They were explained why it is important to understand texts, what main ideas are, and how they can be recognized. Then, they were introduced to the three cognitive text reduction strategies and explained how to use them correctly. Students were also taught about metacognitive strategies (e.g., goal setting and strategy monitoring) and why these strategies are important.

During the baseline week, the students tried out the cognitive and metacognitive strategies. At the start of the week, students set a goal regarding how many of the 10 main ideas in the texts they aimed to identify correctly on average. Then they worked on one expository text per school day (i.e., on five texts) and tried to identify each text’s main ideas using the cognitive strategies that had been introduced to them before the baseline week. Immediately after working on each text, students rated the extent to which they had previously monitored themselves while using their cognitive strategy. Every day, the teachers discussed with the students the text they had last completed and the 10 main ideas that this text contained. Students then recorded how many main ideas they had correctly identified in the respective text.

During the 4-week intervention (the proceduralization weeks), the students systematically practiced and proceduralized the strategies that had been introduced to them before and that they had tried out during the baseline week. In each of the four proceduralization weeks, the students worked on five texts, as they had in the baseline week. As in the baseline week, students set themselves a goal at the beginning of each week, recorded in their learning diary the extent to which they had monitored their cognitive strategy use, corrected each text with their teacher, and recorded how many of its main ideas they had identified correctly. All these processes were supported by guided reflections of the students and extensive feedback from their teachers (see Stoeger et al., 2014, for more information about the intervention and its effectiveness).

### Plan of Analysis

For the first aim of our study, to investigate the relationship between ITs and the use of learning strategies, we analyzed relations between ITs and (a) students’ self-reported use of cognitive and metacognitive learning strategies (replication of existing research) and (b) the behavior-proximal measures of these strategies that had been gathered during the baseline week. For the second aim of our study, to investigate the relationship between ITs and the extent to which students benefit from the 4-week intervention (proceduralization weeks), we analyzed how much students’ strategy use increased, both on the self-report scales and on the behavior-proximal measures.

In order to investigate the questions related to the two aims simultaneously, we calculated mixed linear regression analyses (hierarchical linear models). To test whether students with a stronger incremental theory show higher values for the measures of learning strategy use (first aim), we examined whether ITs can predict the baseline level (intercept) for these measures. To test whether students with a stronger incremental theory profit more from the 4-week intervention on learning strategies (second aim), we looked at whether ITs can predict the rate of growth for these measures. For these analyses, we used R (Version 3.6.3) and its lme4 package (Bates et al., 2015).

Due to the three-level structure of the data (with measurement points nested in students, and students nested in classrooms), we first investigated the extent to which we needed to take this structure into account in the form of including random effects. As all models ended up containing random effects, we estimated the variance explained by fixed effects (*R*^2^_Marginal_) as well as the variance explained by both fixed and random effects (*R*^2^_Conditional_) using the MuMIn package (Barton, 2018).

Because we expected values for learning strategy use (self-report scales as well as behavior-proximal measures) to increase over the course of the learning strategy intervention, all our models included measurement point (i.e., linear change over time) as a predictor for strategy use. The two models predicting the self-report scales for strategy use contain two measurement points each (before and after the intervention; *T* = 0 and *T* = 1); the three models predicting the behavior-proximal measures for strategy use contain five measurement points each (one per week; *T* = 0 to *T* = 4; with *T* = 0 representing the baseline week). We also tested whether the models for behavior-proximal measures of strategy use with their five measurement points had a significant quadratic change component (*T* = 0, *T* = 1, *T* = 4, *T* = 9, and *T* = 16). This was done because students’ increases in effectiveness of learning strategy use might either level off over the course of the intervention (a frequently occurring characteristic of learning trajectories; see Newell and Rosenbloom, 1981; Newell et al., 2001) or accelerate (as is common with learning tasks where the learner must first master the basics before more rapid improvement becomes possible; see Pusic et al., 2015).

Next, we added strength of incremental theory to the models. First, we included the variable in the form of a main effect. This was done to investigate whether students with a stronger incremental theory would already report more learning strategy use before the intervention and whether they would show higher values on the behavior-proximal measures of strategy use during the baseline week. Second, we included strength of incremental theory as part of an interaction effect with measurement point. This was done to investigate whether students with a stronger incremental theory would show greater increases on self-report scales and behavior-proximal measures of strategy use over the course of the intervention.^2^

Finally, we added reading comprehension as a covariate to all models. This was mainly done so that we could use the remaining variation in number of correctly identified main ideas as an indicator for how effectively students used the cognitive strategies. In addition to the main effect of reading comprehension, we also included its interaction effect with the measurement point. This was done to account for differences regarding the amount of increase in strategy use depending on students’ levels of reading comprehension.

## Results

### Preliminary Analyses

First, we assessed the psychometric properties of all variables (see Table 1) and calculated the correlations for each pair of them before the intervention (i.e., for self-report scales of strategy use before the intervention and for behavior-proximal measures of strategy use during the baseline week, see Table 2). Next, we checked the extent to which the three-level structure of the data needs to be considered. This was done by calculating an unconditional random effects model for each indicator of learning strategy use. The variance decomposition for each of these models can be found in Table 3; as most measures of strategy use showed a substantial amount of variance on both level 2 (between students) and level 3 (between classrooms), we included random intercepts for both levels in all of our models.

**Table 1:** Psychometric properties of all variables.

Variable	Indicators	α	*M*	*SD*	Range	Skew	Kurtosis
**Incremental theory**	6	0.68	4.86	0.72	1.83–6.00	−0.69	0.72
**Reading comprehension**	24	0.77	18.36	3.91	2.00–24.00	−1.22	1.56
**Cognitive strategies scale**							
Before strategy introduction	4	0.70	2.75	1.04	1.00–6.00	0.37	−0.45
After intervention	4	0.50	3.69	1.05	1.00–6.00	−0.47	0.13
**Metacognitive strategies scale**							
Before strategy introduction	18	0.84	0.35	0.23	0.00–1.00	0.49	−0.44
After intervention	18	0.93	0.38	0.33	0.00–1.00	0.48	−1.03
**Correctly identified main ideas**							
First week (baseline week)	5	0.78	6.90	1.34	2.20–10.00	−0.82	0.83
Second week	5	0.81	6.48	1.47	0.80–9.80	−0.70	0.69
Third week	5	0.84	7.10	1.64	0.60–10.00	−0.72	0.39
Fourth week	5	0.85	7.11	1.56	0.75–10.00	−0.70	1.05
Fifth week	5	0.82	7.50	1.46	2.20–10.00	−0.78	0.76
**Strategy monitoring**							
First week (baseline week)	5	0.91	4.31	1.31	1.00–6.00	−0.86	0.57
Second week	5	0.94	4.32	1.28	1.00–6.00	−0.93	0.43
Third week	5	0.94	4.39	1.28	1.00–6.00	−0.94	0.48
Fourth week	5	0.96	4.45	1.37	1.00–6.00	−1.21	0.62
Fifth week	5	0.96	4.46	1.37	1.00–6.00	−1.04	0.46
**Deviation from week’s goal**							
First week (baseline week)	1		1.59	1.13	0.00–5.80	0.68	0.13
Second week	1		1.39	1.23	0.00–7.80	1.57	3.34
Third week	1		1.58	1.28	0.00–7.00	1.33	1.33
Fourth week	1		1.38	1.18	0.00–6.00	1.24	1.24
Fifth week	1		1.23	1.10	0.00–7.20	1.63	1.63

**Table 2:** Pearson correlation matrix for all variables before the intervention.

Variable	1	2	3	4	5	6	7
1. Incremental theory	—						
2. Reading comprehension	0.28^**^	—					
3. Cognitive strategies scale	0.02	−0.16^*^	—				
4. Metacognitive strategies scale	0.15^*^	0.03	0.13^*^	—			
5. Correctly identified main ideas	0.29^**^	0.32^**^	−0.01	0.03	—		
6. Strategy monitoring	0.18^**^	−0.02	0.08	0.16^*^	0.15^*^	—	
7. Deviation from week’s goal	−0.08	−0.04	0.08	−0.03	0.06	−0.05	—

* *p* < 0.01;

** *p* < 0.001.

**Table 3:** Variance decompositions for the unconditional random effects models.

Dependent variable	Level 1 (within students)	Level 2 (between students)	Level 3 (between classrooms)
Cognitive strategies scale	91.0%	0.0%	9.0%
Metacognitive strategies scale	53.6%	38.0%	8.4%
Correctly identified main ideas	39.5%	45.5%	14.9%
Strategy monitoring	25.5%	67.2%	7.3%
Deviation from week’s goal	80.8%	16.2%	3.0%

We then tested for each model whether there was a significant linear change component over the course of the intervention, and included this component if it was significant. In addition, for the three models predicting the behavior-proximal measures of strategy use (five measurement points), we also tested whether there was a significant quadratic change component, and included this component if it was significant. Next, we tested whether model fit could be improved by adding random slopes for measurement point (i.e., by allowing rates of change to differ between students and/or classrooms). As a result of this, we included random slopes (a) on level 2 for the linear change component in the models for the number of correctly identified main ideas, strategy monitoring, and deviation from week’s goal; (b) on level 3 for the linear change component in the models for the cognitive strategies scale, for the number of correctly identified main ideas, and deviation from week’s goal, and (c) on level 2 for the quadratic change component in the model for number of correctly identified main ideas. Finally, we included both strength of incremental theory and reading comprehension in each of the models, followed by including the interaction effects between (a) strength of incremental theory and linear change and (b) reading comprehension and linear change.

### Mixed Linear Regression Analyses

#### Predicting Learning Strategy Use

To address the first aim of our study, we examined whether students’ ITs predict their use of cognitive and metacognitive strategies, assessed with (a) self-report scales and (b) behavior-proximal measures. The final mixed linear regression models for predicting the self-report scales (while controlling for reading comprehension) can be found in Table 4. Contrary to our expectations, strength of incremental theory was unrelated to cognitive strategy use measured by the self-report scale before the intervention (*b* = 0.03, *p* = 0.549). Yet as expected, strength of incremental theory was positively related to metacognitive strategy use measured by the self-report scale before the intervention (*b* = 0.03, *p* = 0.021).

**Table 4:** Linear mixed-effects models predicting the self-report scales for learning strategy use.

Predictor	*b*	*SE*	*df*	*t*	*p*
**Cognitive strategies scale; *R*^2^_Marginal_ = 0.191; *R*^2^_Conditional_ = 0.541**					
γ_00_	2.74	0.13	19.0	20.75	<0.001
Linear change	0.96	0.15	19.1	6.48	<0.001
Incremental theory	0.03	0.05	776.9	0.60	0.549
Reading comprehension	−0.18	0.05	782.1	−3.60	<0.001
Linear change × Incremental theory	−0.01	0.06	422.9	−0.23	0.815
Linear change × Reading comprehension	0.05	0.06	426.7	0.90	0.369
**Metacognitive strategies scale; *R*^2^_Marginal_ = 0.011; *R*^2^_Conditional_ = 0.470**					
γ_00_	0.35	0.02	22.1	15.62	<0.001
Linear change	0.03	0.01	431.8	2.21	0.028
Incremental theory	0.03	0.01	716.4	2.31	0.021
Reading comprehension	0.00	0.01	705.7	0.19	0.850
Linear change × Incremental theory	−0.02	0.01	431.6	−1.15	0.252
Linear change × Reading comprehension	−0.01	0.01	431.5	−0.67	0.501

*R*^2^_Marginal_ = Variance explained by fixed effects; *R*^2^_Conditional_ = Variance explained by both fixed and random effects. Both incremental theory and reading comprehension were *z*-standardized before the analyses.

The final models for predicting the three behavior-proximal measures of strategy use (while controlling for reading comprehension) can be found in Table 5. As expected, strength of incremental theory was positively related to the number of correctly identified main ideas during the baseline week (*b* = 0.32, *p* < 0.001). Also, as expected, strength of incremental theory was positively related to the amount of strategy monitoring during the baseline week (*b* = 0.18, *p* = 0.002). Yet unexpectedly, strength of incremental theory was not significantly related to students’ effectiveness of goal setting (i.e., how much the number of main ideas correctly identified in the daily texts during the baseline week deviated from the goals they had set at the beginning of that week; *b* = −0.06, *p* = 0.189).

**Table 5:** Linear mixed-effects models predicting the behavior-proximal measures of strategy use.

Predictor	*b*	*SE*	*df*	*t*	*p*
**Correctly identified main ideas; *R*^2^_Marginal_ = 0.136; *R*^2^_Conditional_ = 0.699**					
γ_00_	6.79	0.11	18.8	63.46	<0.001
Linear change	−0.10	0.05	235.6	−1.94	0.054
Quadratic change	0.07	0.01	424.7	6.31	<0.001
Incremental theory	0.32	0.06	424.6	5.30	<0.001
Reading comprehension	0.34	0.06	429.4	5.42	<0.001
Linear change × Incremental theory	−0.04	0.02	411.4	−2.26	0.025
Linear change × Reading comprehension	0.01	0.02	397.1	0.56	0.578
**Strategy monitoring; *R*^2^_Marginal_ = 0.019; *R*^2^_Conditional_ = 0.811**					
γ_00_	4.31	0.08	20.2	50.94	<0.001
Linear change	0.04	0.01	427.4	3.05	0.002
Incremental theory	0.18	0.06	436.6	3.14	0.002
Reading comprehension	−0.08	0.06	430.8	−1.33	0.185
Linear change × Incremental theory	−0.01	0.01	433.7	−0.36	0.718
Linear change × Reading comprehension	−0.01	0.01	435.5	−0.56	0.575
**Deviation from week’s goal; *R*^2^_Marginal_ = 0.017; *R*^2^_Conditional_ = 0.235**					
γ_00_	1.58	0.09	18.8	18.43	<0.001
Linear change	−0.08	0.03	19.9	−2.38	0.027
Incremental theory	−0.06	0.05	625.3	−1.31	0.189
Reading comprehension	−0.09	0.05	648.3	−1.71	0.087
Linear change × Incremental theory	−0.00	0.02	1317.9	−0.22	0.827
Linear change × Reading comprehension	0.01	0.02	1289.5	0.39	0.699

*R*^2^_Marginal_ = Variance explained by fixed effects; *R*^2^_Conditional_ = Variance explained by both fixed and random effects. Both incremental theory and reading comprehension were *z*-standardized before the analyses.

#### Increases in Learning Strategy Use

**Figure 1:**
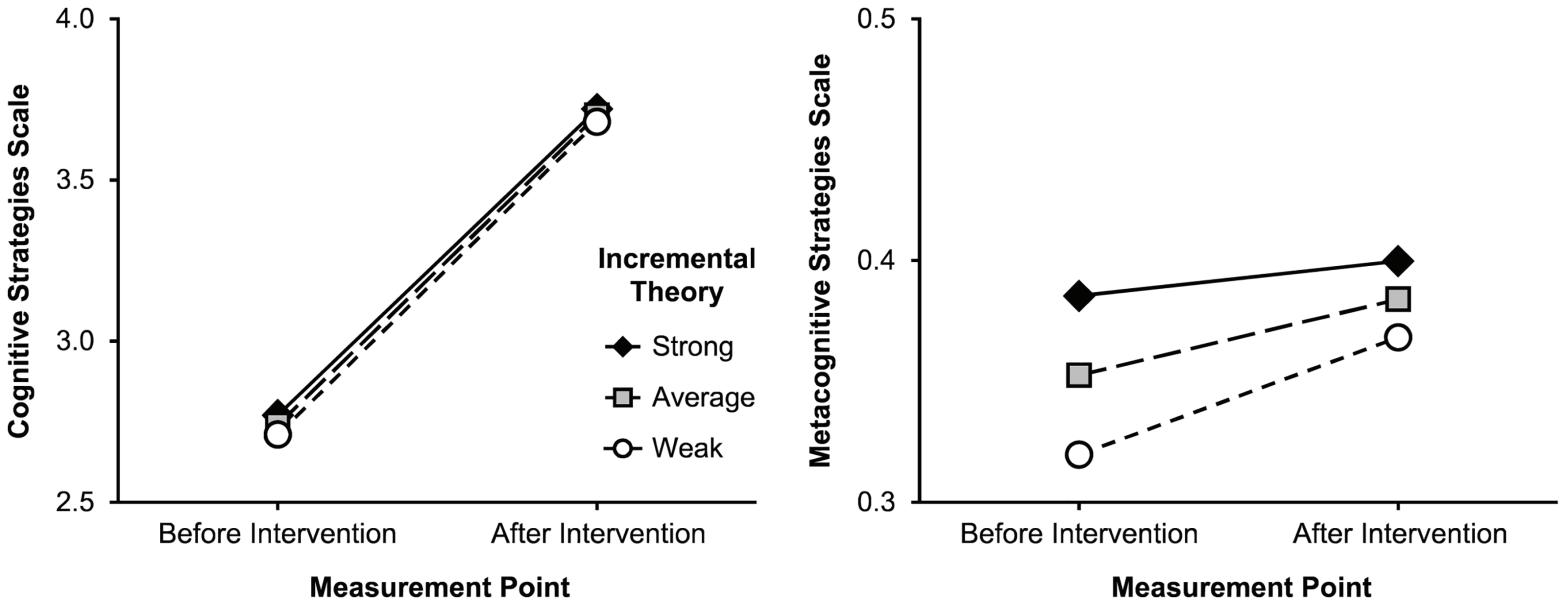
Predicted values for self-report scales of learning strategy use. The measures were predicted before and after the learning strategy intervention for different strengths of incremental theory (strong = one *SD* above the mean, weak = one *SD* below the mean). Values are based on the models from Table 4 that control for reading comprehension.

**Figure 2:**
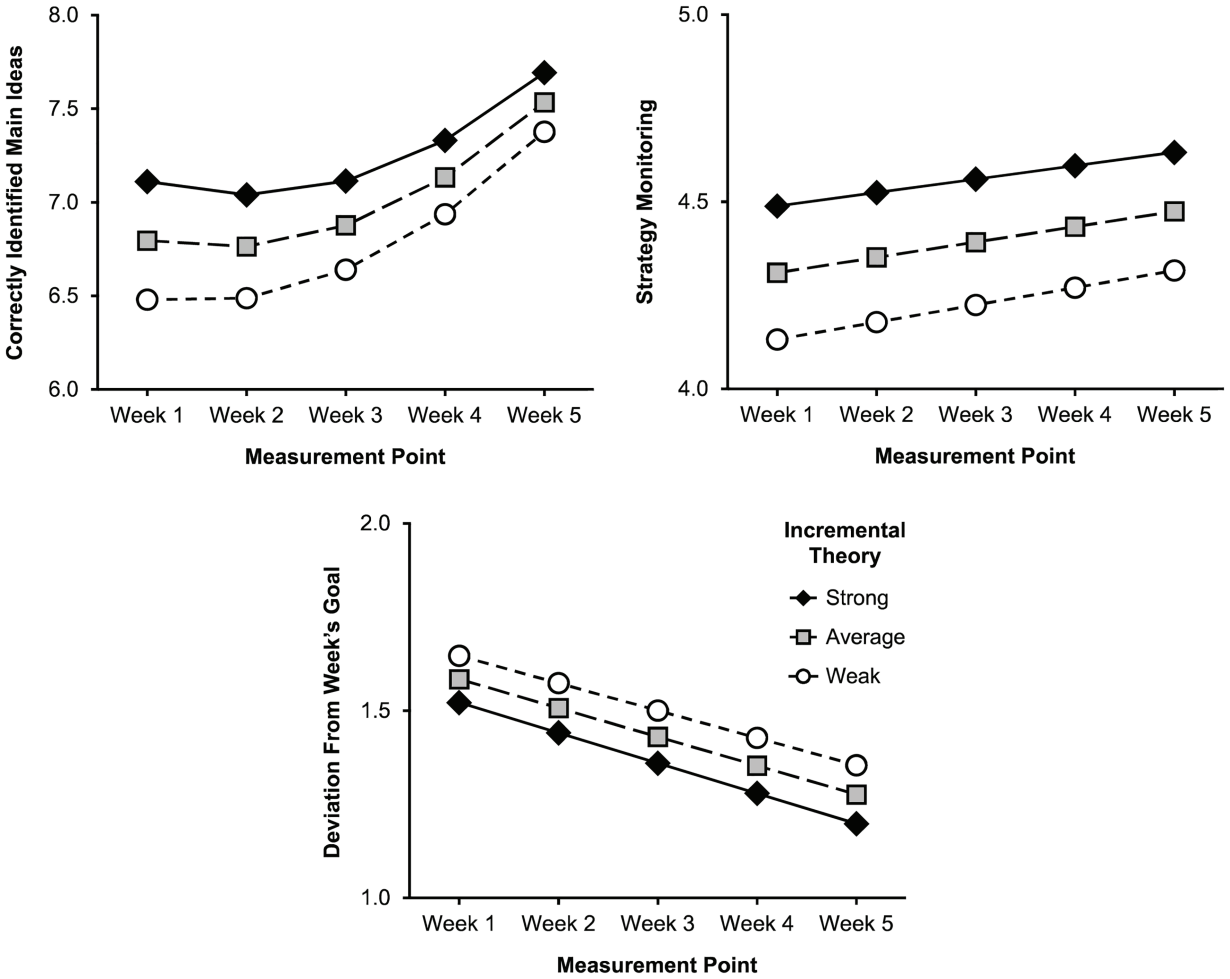
Predicted values for behavior-proximal measures of learning strategy use. The measures were predicted for the baseline week (week 1) and the four proceduralization weeks (weeks 2–5) for different strengths of incremental theory (strong = one *SD* above the mean, weak = one *SD* below the mean). Values are based on the models from Table 5 that control for reading comprehension.

Before addressing the second aim of our study, we examined whether self-reported and behavior-proximal measures of learning strategy use actually increased over the course of the intervention. We found that this was the case for both the self-report scales (see Figure 1 for plots) and all behavior-proximal measures (see Figure 2 for plots). There was a significant positive linear change component for both the self-report scale for cognitive strategy use (*b* = 0.96, *p* < 0.001) and the one for metacognitive strategy use (*b* = 0.03, *p* = 0.028) from before to after the intervention.

For the behavior-proximal measure of cognitive learning strategies (correctly identified main ideas), there was no significant linear change component (*b* = −0.10, *p* = 0.054), but a significant quadratic change component (*b* = 0.07, *p* < 0.001), signifying an increasing rate of growth from the baseline week (week 1) over the course of the 4-week intervention. For the first behavior-proximal measure of metacognitive strategy use, monitoring, we found a significant positive linear change component (*b* = 0.04, *p* = 0.002). For the second behavior-proximal measure of metacognitive strategy use, deviation from week’s goal, there was, as expected, a significant negative linear change component (*b* = −0.08, *p* = 0.027), signifying that goal setting became increasingly realistic from the baseline week (week 1) over the course of the four proceduralization weeks. Thus, cognitive and metacognitive learning strategy use increased over the course of the intervention.

#### Predicting Growth Rates for Learning Strategy Use

To address the second aim of our study, we investigated whether ITs predict the extent to which students benefit from the intervention, that is, whether ITs predict rates of growth for the measures of cognitive and metacognitive strategy use. Here, none of our hypotheses were supported. Contrary to our expectations, there was no interaction effect between strength of incremental theory and linear change for either the self-report scale for cognitive strategy use (*b* = −0.01, *p* = 0.815) or the one for metacognitive strategy use (*b* = −0.02, *p* = 0.252). Also, unexpectedly, strength of incremental theory was negatively related to linear change in number of correctly identified main ideas from the baseline week (week 1) over the course of the four proceduralization weeks (*b* = −0.04, *p* = 0.025), signifying a smaller growth rate for students with a stronger incremental theory. Moreover, there was no interaction effect between strength of incremental theory and linear change for either strategy monitoring (*b* = −0.01, *p* = 0.718) or deviation from week’s goal (*b* = −0.00, *p* = 0.827).

## Discussion

This study had two aims. The first aim was to investigate the relationship between third and fourth graders’ implicit theories about ability and their use of cognitive and metacognitive learning strategies, assessed with (a) typical broad-brush self-report scales and (b) behavior-proximal measures. The second aim was to investigate the relationship between implicit theories and the extent to which these students benefit from an intervention on cognitive and metacognitive learning strategies, that is, whether students with a more incremental theory show greater increases on self-report scales and behavior-proximal measures for cognitive and metacognitive strategy use when they participate in a 4-week intervention. These measures were collected in the context of authentic academic learning situations.

With respect to the first aim, our predictions regarding the self-report scales were only partially confirmed. The prediction that children with a more incremental theory would report using more learning strategies was not supported for the self-report scale for cognitive strategies. However, it was supported for the self-report scale for metacognitive strategies.

The fact that we found no relationship between ITs and the self-report scale for cognitive strategy use was somewhat surprising in light of existing research. Several studies document the relationship we had expected for adult learners (Bråten and Olaussen, 1998; Martin et al., 2001; Mega et al., 2014; Yan et al., 2014; Karlen and Compagnoni, 2017), high school students (Ommundsen, 2003; Ommundsen et al., 2005; Martin et al., 2013), and primary school students (Stipek and Gralinski, 1996; Law, 2009). One possible explanation for our null finding is that a large proportion of the children apparently had little experience with the specific cognitive strategies under investigation (that are focused on extracting a text’s main ideas) at the first measurement point: the average value for the items querying cognitive strategy use was only 2.75, a value that can be located slightly below the response option *somewhat disagree* (value 3). It seems likely that most children who were not familiar with these strategies reported not using them, irrespective of how much they held an incremental theory, thereby weakening the relationship between ITs and the self-report scale for cognitive strategy use.

The finding that children with a stronger incremental theory showed higher values on the self-report scale for metacognitive strategy use, however, is in line with existing studies. Similar results have been obtained in numerous investigations with adult learners (Bråten and Olaussen, 1998; Vermetten et al., 2001; Dupeyrat and Mariné, 2005; Mega et al., 2014; Stump et al., 2014) and in some studies with high school students (Stipek and Gralinski, 1996; Ommundsen, 2003). However, we are aware of only one study that has investigated this relationship with younger learners (Law, 2009). Thus, our study contributes additional evidence that the positive relationship between holding more of an incremental theory about one’s ability and reporting to use more metacognitive strategies also exists for younger students (i.e., third and fourth graders). Demonstrating this is important partly because the relationship between ITs and the beliefs typically associated with them (e.g., beliefs about the meaning of effort) is still in the process of solidifying at the end of the primary school years (see Dweck, 2002).

For the behavior-proximal measures of cognitive and metacognitive learning strategy use, our predictions regarding the first aim of our study were mostly confirmed: children with a more incremental theory showed greater values for learning strategy use and effectiveness on most of these measures. Our predictions were supported for cognitive strategy use, operationalized in the form of correctly identified main ideas: children with a more incremental theory correctly identified more main ideas in the five texts of the baseline week (even when controlling for reading comprehension, as we did in all models). With regard to metacognitive strategy use, children with a more incremental theory also monitored their learning behavior more while using the cognitive strategies. However, the second behavior-proximal measure of metacognitive strategy use, realistic goal setting, was unrelated to strength of incremental theory.

The finding that children with a stronger incremental theory showed higher values for our behavior-proximal measure of cognitive learning strategy use adds to the existing literature. We are aware of only one other study that has investigated the relationship between ITs and such a behavior-proximal measure (Greene et al., 2010)—that, in contrast to our study, has obtained a null result. Thus, our investigation complements the aforementioned studies that have used self-report scales and provides initial evidence that the positive relationship between strength of incremental theory and cognitive strategy use still holds when behavior-proximal measures of strategy use are employed in an ecologically valid setting.

The findings regarding the relation between strength of children’s incremental theory and the behavior-proximal measures of metacognitive learning strategy use, namely monitoring and goal setting, were mixed. During the baseline week, the stronger students’ incremental theory was, the more they monitored their strategies while working on the week’s texts. There was, however, no relationship between strength of incremental theory and how realistic students’ goals were. A possible explanation for this null result could be that during the baseline week, most children were probably not yet familiar with the task of setting realistic goals, resulting in children with a more incremental mindset performing no better than children with a less incremental mindset.

Our findings on the relationship between ITs and the behavior-proximal measures of metacognitive learning strategy use are new in several respects. First, all earlier studies investigating ITs in combination with such behavior-proximal measures we found were conducted with adult learners. In contrast, our study was conducted with primary school students. Second, only few studies have investigated these relationships with a focus on learning situations that one might encounter in an academic context (Greene et al., 2010; Karlen and Compagnoni, 2017). Our study contributes to this literature by employing behavior-proximal measures of metacognitive strategy use in a school-related setting where students worked on authentic learning tasks by trying to identify the main ideas in expository texts. Third, by assessing the extent to which children monitored their strategy and set realistic goals, our study sheds light on aspects of metacognitive strategy use that had hitherto, to our knowledge, not been investigated in combination with ITs. Previous studies with non-academic laboratory tasks that assessed indicators for the use of specific metacognitive strategies have found that participants who held more of an incremental theory set more challenging goals (Wood and Bandura, 1989; Tabernero and Wood, 1999; Beckmann et al., 2012). The two studies investigating overall indicators of metacognitive strategy use in learning situations that might be found in academic contexts have obtained null results (Greene et al., 2010, coded participants’ verbalizations during a learning task, while Karlen and Compagnoni, 2017, coded participants’ open-ended responses about what they did before, during, and after writing an academic essay).

Our predictions regarding the second aim of our study were not supported for any of the learning strategy measures under investigation. We had expected that children with a stronger incremental theory would benefit more from an intervention on cognitive and metacognitive learning strategies, that is, that their indicators of strategy use would increase more over the course of the intervention. This was not the case: neither did children with a more incremental theory show greater increases on the self-report scales for cognitive or metacognitive learning strategy use, nor did they show greater increases on the behavior-proximal measures for such strategies. Although all indicators for students’ use of cognitive and metacognitive learning strategies increased over the course of the intervention, the size of the change was, for almost all of the indicators, unrelated to children’s ITs. The only exception was the unexpected negative relationship between strength of incremental theory and rate of growth for the behavior-proximal measure of cognitive learning strategies (i.e., the weekly number of correctly identified main ideas) that was present although we had controlled for reading comprehension, indicating that gains in effectiveness of cognitive strategy use might have leveled off more quickly for children who held more of an incremental theory.

These findings may seem surprising at first because existing literature suggests that compared to entity theorists, incremental theorists are more likely to be oriented toward enhancing their competencies (see Burnette et al., 2013) and more likely to seize learning opportunities (Hong et al., 1999; Nussbaum and Dweck, 2008), which should also help them to make better use of a learning strategy intervention. One possible explanation for our findings could be the fact that the intervention strongly emphasized an individual reference norm (see Stoeger et al., 2014), which might have eclipsed the effect of ITs: the children were taught that they could all improve their learning strategies and thus their performance in identifying main ideas—regardless of their baseline levels. Perhaps this message, combined with the intervention’s daily systematic feedback on learning gains, was so compelling that even children without an incremental theory were persuaded to fully engage with the strategies.

Particularly unexpected was the negative relationship between strength of incremental theory and rate of growth for correctly identified main ideas. The more children held an incremental theory, the weaker their improvements in performance were. This result might have to do with the fact that strength of incremental theory predicted higher values for correctly identified main ideas during the baseline week, thus making further increases in performance over the course of the proceduralization weeks less likely.

### Limitations and Future Research

Although our investigation largely replicates the findings of previous studies and broadens the research on the relationships between ITs and the use of learning strategies, it also has several limitations. A first limitation concerns our behavior-proximal measures of strategy use. Although the measures of metacognitive strategy use are closer to actual learning behavior than the self-report scales employed in most studies that investigate ITs and learning strategy use, they nevertheless prompt participants to report their amount of strategy monitoring and their self-set goals. One might argue that students’ responses to being prompted to record a goal at the beginning of each week and to report their amount of strategy monitoring after having worked on the respective text might differ markedly from responses to less reactive measures of goal setting and monitoring. Also, the number of correctly identified main ideas cannot be considered a pure measure for effectiveness of cognitive strategy use, but is likely to also depend on students’ general ability to identify main ideas—although controlling for reading comprehension should have alleviated this problem. Nevertheless, we might have obtained a purer measure if we had also controlled for students’ general cognitive ability. To provide even more robust measures of learning strategy use, further studies could code students’ verbalizations during a learning task (as Greene et al., 2010, have done), or covertly collect trace data of students’ strategy use while they work in a virtual learning environment.

A second limitation lies in the young age of the participants and the fact that the sample comes from a somewhat special population (i.e., primary school students from Bavaria, Germany). Both sampling circumstances raise the question as to which extent the results can be generalized to older students and students from other populations (e.g., to high school students from the United States or Japan). At the end of German primary school (i.e., at the end of fourth grade), many students do not yet seem to have a particularly strategic approach to learning (Sontag et al., 2012), which might attenuate the relationships between learning strategy use and related constructs such as ITs. Thus, stronger relationships might be found, for example, in higher grades or more challenging school systems. Also, in this age group in general, many students tend to hold more of an incremental theory rather than an entity theory (see Dweck, 2002), which is in line with the rather high values for incremental theory that we observed in our study. Therefore, the relationship between ITs and learning strategy use might be somewhat weaker for such young students because older students might show greater variance in strategic learning and ITs due to the increasing academic demands during secondary education. Thus, further studies could investigate students from different school systems and focus on grades 5 and 6—another age group in which the relationship between ITs and learning strategy use has hardly been investigated.

A final limitation lies in the fact that the design of our study does not allow conclusions to be drawn about the directions of influence between the variables under investigation. For example, it is also plausible that frequent use of cognitive and metacognitive learning strategies might lead learners to develop a more incremental theory. If learners use more effective learning strategies, they are more likely to realize that their strategic approach greatly influences how successful they are (see Zimmerman, 2000, 2008)—and that success depends on more than just innate abilities. To allow for stronger conclusions, further studies could directly manipulate ITs. This might be done by letting half of the sample take part in an intervention that teaches an incremental theory (like the one described in Paunesku et al., 2015) before these students participate in a learning strategy intervention, and then investigating whether this IT intervention affects actual and effective strategy use and the development of strategy use over time.

### Conclusion

In summary, our study provides initial evidence that the positive relationship between having more of an incremental theory and reporting to use more metacognitive learning strategies can be generalized to younger students in an ecologically valid setting. The study also provides some initial evidence that having more of an incremental theory predicts more actual and effective use of cognitive and metacognitive strategies for this age group. Although further studies are needed to provide more causal evidence, our findings are consistent with the idea that ITs are already related to learning behavior at the end of the primary school period—despite the fact that the network of beliefs associated with ITs has not yet fully solidified for most students at that point (see Dweck, 2002). Further studies about the relationship between ITs and learning strategy use with younger students could apply less reactive measures of strategy use (e.g., students’ verbalization or trace data), investigate a slightly older sample of students in more challenging learning settings, and attempt to influence students’ ITs directly and investigate the effect of such changes on learning strategy use.

In terms of practical recommendations regarding ITs and learning strategies, interventions that influence ITs (targeting motivational aspects of learning) and interventions that teach learning strategies (targeting strategic aspects of learning) might have potential for complementing each other—especially when aimed at students at the end of primary school: Since learning strategies should already be taught during the primary school period (see Dignath and Büttner, 2008), and since ITs and other learning-related and motivational beliefs are still taking shape during that time period (see Barger and Linnenbrink-Garcia, 2016), it seems appropriate to address strategies and beliefs together. When considering learning strategy interventions, it is important to note that students’ effective use of strategies strongly depends on motivational characteristics, such as interest, confidence in one’s own competencies, and the desire to improve one’s own abilities (see Pintrich, 2000; Zimmerman and Schunk, 2008). However, all these aspects of motivation tend to decrease over the course of students’ school careers (Anderman and Midgley, 1997; Gottfried et al., 2001; Fredricks and Eccles, 2002; Jacobs et al., 2002; Lepper et al., 2005; Bong, 2009), accompanied by an increase in the prevalence of an entity mindset (see Dweck, 2002). Thus, an IT intervention (like the intervention confirmed as effective in Yeager et al., 2016) might improve the effectiveness and sustainability of learning strategy interventions, also and perhaps especially for older students. When considering IT interventions, it is important to note that merely teaching IT-related beliefs conducive to learning (i.e., that abilities can be substantially increased, that effort signifies optimal challenges, and that setbacks are learning opportunities) may often not be sufficient for learners to achieve their goals. This can result in frustration and demotivation in the long run unless learners are also taught the strategies necessary to achieve these goals (see Dweck and Yeager, 2019). Thus, a learning strategy intervention might improve the effectiveness and sustainability of an IT intervention by supplying learners with the strategies they need to translate their increased effort and desire to learn into achievement.

## Data Availability Statement

The datasets presented in this article are not readily available because of local data protection regulations. Requests to access the datasets should be directed to benjamin.matthes@ur.de.

## Ethics Statement

Ethical review and approval was not required for the study on human participants in accordance with the local legislation and institutional requirements. Written informed consent to participate in this study was provided by the participants’ legal guardian/next of kin.

## Author Contributions

BM and HS conceptualized the study. BM conducted the study, analyzed the data, and wrote the first draft of the manuscript. HS supervised the study and provided feedback. All authors contributed to the article and approved the submitted version.

### Conflict of Interest

The authors declare that the research was conducted in the absence of any commercial or financial relationships that could be construed as a potential conflict of interest.

## Footnotes

^1^Two teachers did not implement the intervention regularly. Two other teachers did not implement the intervention in its entirety, with one of them not implementing the last proceduralization week and another one completing only four of the five texts per week with her students.

^2^Whereas the widely-used software HLM (Raudenbusch et al., 2011) tests the effect of a predictor on rate of change if the user includes the predictor in the respective level-2 equation, the lme4 package instead requires the user to include an interaction effect between the predictor and the measurement point in the model.
